# Imaging cardiac SCN5A using the novel F-18 radiotracer radiocaine

**DOI:** 10.1038/srep42136

**Published:** 2017-02-16

**Authors:** Jacob M. Hooker, Martin G. Strebl, Frederick A. Schroeder, Hsiao-Ying Wey, Amrut V. Ambardekar, Timothy A. McKinsey, Matthias Schoenberger

**Affiliations:** 1 Harvard Medical School, Massachusetts General Hospital, Martinos Center for Biomedical Imaging, 149 13th Street, 02129 Charlestown, MA, USA; 2 Department of Medicine, Division of Cardiology and Consortium for Fibrosis Research and Translation, University of Colorado – Anschutz Medical Campus, Aurora, CO, USA

## Abstract

The key function of the heart, a well-orchestrated series of contractions, is controlled by cardiac action potentials. These action potentials are initiated and propagated by a single isoform of voltage gated sodium channels – SCN5A. However, linking changes in SCN5A expression levels to human disease *in vivo* has not yet been possible. Radiocaine, an F-18 radiotracer for positron emission tomography (PET), is the first SCN5A imaging agent in the heart. Explants from healthy and failing human hearts were compared using radiocaine autoradiography to determine that the failing heart has ~30% lower SCN5A levels - the first evidence of changes in SCN5A expression in humans as a function of disease. Paving the way for translational imaging, radiocaine proved to exhibit high *in vivo* specific binding to the myocardium of non-human primates. We envision that SCN5A measurements using PET imaging may serve as a novel diagnostic tool to stratify arrhythmia risk and assess for progression of heart failure in patients with a broad spectrum of cardiovascular diseases.

Voltage gated sodium channels (NaVs) comprise a family of nine transmembrane proteins (NaV1.1–NaV1.9) that conduct sodium currents across membranes in response to changes in membrane voltage^1^. As such, they play the critical step in fast electrical communication by initiating and propagating action potential firing^2^. While neurons of the central and peripheral nervous system are equipped with different populations of NaVs, there is one single isoform, NaV1.5 (SCN5A) dominating the myocardium^3^. SCN5A is the initiator of cardiac electrical signaling, and thus controls heart rate and contraction. Malfunction of SCN5A and changes in heart sodium current has severe consequences^4^,^5^,^6^. For instance, mutations of the *SCN5A* gene underlie the lethal arrhythmias seen in long-QT-syndrome and Brugada syndrome^7^,^8^. In addition, reduced SCN5A expression has been linked to disease progression in a mouse model of dilated cardiomyopathy (DCM) and may contribute to the conduction disturbances observed in human heart failure^9^,^10^. Furthermore, modulation of the sodium channel may have a therapeutic effect as class I antiarrhythmatics such as lidocaine (class Ib) and flecainide (class Ic) target the sodium channel and are used clinically to prevent ventricular fibrillation and tachycardia^11^,^12^.

Although SCN5A is critically important in cardiac function, there is a lack of knowledge about its expression density in healthy individuals and as a function of disease. For example, current methods do not enable measurement of SCN5A density in patients to gauge response to therapies such as antiarrhythmic agents or cardiac pacemakers/defibrillators, and it is not possible to quantify drug occupancy levels at SCN5A that are efficacious. Finally, *in vivo* SCN5A density measurement may serve as a novel diagnostic and prognostic tool in the stratification of arrhythmia and heart failure risk, but we are unable to measure it in the living human heart. To fill these gaps in knowledge we have developed a positron emission tomography (PET) radiotracer that engages SCN5A in the myocardium.

## Methods

All methods including living animals or tissue samples from humans and animals were carried out in accordance with relevant guidelines and regulations. All experimental protocols were approved by the Institutional Animal Care and Use Committee (IACUC) of Massachusetts General Hospital (MGH). Informed consent was received from all human tissue donors. The Colorado Multicenter Institutional Review Board approved the protocol for the collection, storage, and analysis of human tissue.

Statistical analysis was performed using Microsoft excel or GraphPad Prism. Capital “N” refers to the number of animals or subjects, lower case “n” refers to the number of repeats.

### *Ex vivo* Autoradiography (standard protocol)

Twenty μm thick heart sections were cut using a −20 °C cryostat and thaw-mounted onto gelatin-coated slides. These slides were stored at −20 °C until the day of the experiment. Sections were then incubated at room temperature in a 50 mL baths containing either 10 mM Tris-HCl or 10 mM Tris-HCl and the indicated concentration of lidocaine or fluorolidocaine for 10 min. Radiocaine was prepared as described in the Supplementary Information, and 100 μCi of the radiotracer were added to each bath. Following 15 min incubation at room temperature, sections were dipped 3× in a fresh bath containing 10 mM Tris-HCl and subsequently washed for 1 min in an additional bath of 10 mM Tris-HCl. Slides were carefully wiped dry on absorbent towels, dried in vacuum for 30 min and exposed for 1 h to a multisensitive phosphorscreen. Phosphorscreens were developed using a Cyclone Plus phosphorimager (both from PerkinElmer) and the resulting parent image was evaluated using ImageJ software (NIH). Individual images of sections were cropped using ImageJ with no additional adjustment to color levels/thresholds. The experimental setup was designed in such a way that both baseline and competition experiments contained the same amount of radioactivity and were imaged on the same screen. For variation of incubation times, the washing time was held constant at 1 min. For variation of washing times, the incubation was held constant at 15 min.

### Rodent PET-CT or PET Data Acquisition and Processing

Male Sprague−Dawley rats (400–500 g) were anesthetized with inhalational isoflurane (Forane) (3% in a carrier of 1.5–2 L/min medical oxygen for induction and 2% isoflurane for maintenance of anesthesia during the scan). Lateral tail vein catheters were placed for i.v. injection of drugs and radiotracer. Two rats were arranged head-to-head in a Triumph Trimodality PET/CT/SPECT scanner (Gamma Medica, Northridge, CA) or a MicroPET P4 scanner. Rats were injected with lidocaine dissolved in saline or pure saline 5 minutes before the start of PET acquisition and radiotracer injection. Dynamic PET acquisition lasted for 45 min and was followed by computed tomography (CT) for anatomic coregistration and attenuation correction. PET data were reconstructed using a 3D-MLEM method resulting in a full width at half-maximum (fwhm) resolution of 1 mm. Reconstructed images were exported from the scanner in DICOM format along with an anatomic CT for rodent studies. These files were imported to AMIDE software and Gaussian filtered (kernel size = 15, fwhm = 1.5 mm). Regions of interest (ROIs) were drawn manually at the lung and myocardium guided by high-resolution CT structural images and summed PET data. TACs were exported in terms of decay corrected activity per unit volume at specified time points with gradually increasing intervals.

### PET/MR Imaging

Two female baboons (Papio anubis, weight = 15.2 ± 1.6 kg) were deprived of food for 12 h prior to the study. Anesthesia was induced with intramuscular ketamine (10 mg/kg) and xylazine (0.5 mg/kg). After endotracheal intubation, the baboon was catheterized antecubitally for radiotracer injection. Anesthesia was maintained using isoflurane (1–1.5%, 100% oxygen, 1 L/min) during the scan, and ketamine/xylazine effects were reversed with yobine (0.11 mg/kg, i.m.) before image acquisition. Vital signs, including heart rate, respiration rate, blood pressure, O_2_ saturation, and end tidal CO_2_, were monitored continuously and recorded every 15 min. Simultaneous PET/MR data were acquired using a Siemens Biograph mMR system (Siemens Healthcare, Erlangen, Germany). Each animal underwent a baseline and a blocking scan on two separate days. MR body imaging was performed with real-time respiratory bellow gating and using the body matrix coil and the built-in spine coil as the receiving coil elements. High-resolution anatomical T1-weighted, dual echo, gradient echo sequence was acquired with the following parameters: TR = 194 ms, TE1/TE2 = 1.23/2.46 ms, matrix size 256 × 256, FOV = 35 cm, phase FOV 65.6% (in-plane resolution = 1.4 mm), 4 mm slice thickness, and 80 slices. For the purpose of MR-based attenuation correction of the PET data, a T1-weighted, 2-point Dixon 3D volumetric interpolated breath-hold examination (VIBE) scan was obtained. PET data were obtained using a single-bed position with an axial field of view of 25.8 cm, transverse field of view of 59.4 cm. PET data were acquired dynamically for 60 min (bolus injection of radiotracer) or 90 min (bolus/infusion of radiotracer) after intravenous administration of [18 F]Radiocaine (5.0 ± 0.2 mCi bolus or 4 mCi bolus  + 4 mCi infusion). PET data were stored in list mode, and reconstruction was performed using a 3D-OSEM method with detector efficiency, decay, dead time, attenuation, and scatter corrections applied. ROIs of the left myocardium and the ventricle were manually delineated from the T1-weighted anatomical image as well as summed PET-images using AMIDE or PMOD® to plot time-activity curves of the myocardium and cardiac blood pool. Standard uptake unit (SUV) was calculated as the mean radioactivity per injected dose per weight.

## Results and Discussion

### NaV-Radiotracer design

Lidocaine has been used in clinical medicine for over half a century and there are more than 300 lidocaine-containing products on the market^13^,^14^,^15^. Lidocaine exhibits micromolar affinity for SCN5A and provided a structural basis for PET radiotracer development. Typically, PET radiotracer design is biased toward higher affinity (low nM) compounds in order to adequately provide signal to background contrast, in particular for low-density targets^16^. However, we recognized that low affinity could be advantageous for SCN5A for two primary reasons: (1) SCN5A density is high in the myocardium and (2) faster dissociation kinetics would accelerate reaching binding equilibrium conditions. As a consequence, signal to background ratios should be sufficient and scan times should be short for a lower affinity ligand. In addition, a lidocaine-based structure would facilitate translation to humans for safety reasons and would have direct relevance to treatment of ventricular arrhythmias. Finally, the choice of a general NaV-blocker such as lidocaine, while providing an SCN5A signal in the myocardium, could serve as a platform technology by opening the door for imaging additional NaV-isoforms in other tissues such as the central and peripheral nervous system.

Bearing in mind a future translational application, we used the isotope F-18, and designed a fluorine-containing lidocaine derivative following established structure activity relationships, which point out tolerance for lipophilic substituents at the terminal ethyl functional groups without affecting affinity^17^. This yielded fluorolidocaine (Figure S1) and its radioactive F-18 analog [^18^F]-fluorolidocaine, which we call *radiocaine* (Figures S1 and S2).

### Radiocaine autoradiography in rats

**Figure 1:**
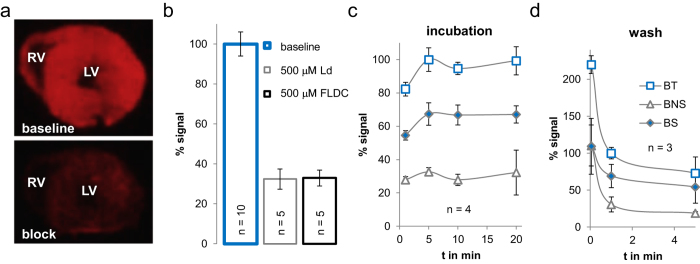
*Ex vivo* imaging using radiocaine autoradiography. (**a**) Images of rat myocardial tissue slices (20 μm) incubated with radiocaine (top) or radiocaine and 500 μM fluorolidocaine (bottom). Both the left and right ventricle (LV & RV) are visible and surrounded by the myocardium, which displays a strong radioactive signal. Suppression of this signal by coincubation of fluorolidocaine demonstrates displaceable binding. (**b**) Comparison of the known SCN5A blocker lidocaine (Ld) and fluorolidocaine (FLDC) as competition ligands at the same concentration. Both ligands lead to the same level of non-displaceable binding. (**c**) Incubation times of 1, 5, 10 and 20 min were tested to investigate equilibrium conditions. After 5 min of radiocaine incubation, specific binding (B_S_) reached a constant level (B_S_ = total binding B_T_ − nonspecific binding B_NS_). (**d**) Washing times of 3 secs, 1 min and 5 min were tested to optimize signal to background conditions and investigate dissociation time course. Wash times of only 1 min are sufficient to reduce non-specific binding to a constant level. (error bars are presented as ± one standard deviation (stdev)).

Using *ex vivo* imaging by means of F-18 autoradiography, we determined the extent of radiocaine specific binding in the rat myocardium and measured its association and dissociation time course. Figure 1a depicts images of a healthy rat myocardial slices (20 μm) incubated with radiocaine (top) or radiocaine with excess non-radioactive fluorolidocaine (bottom) for self-competition. The upper image shows a strong and homogeneous signal across the entire myocardium of the right and left ventricle (RV & LV) as would be expected for SCN5A distribution. The competition experiment shown in the lower image demonstrated that nearly all of the observed signal was saturable and specific binding (B_S_) accounted for ~70% of overall binding (Fig. 1b). A comparison between lidocaine and fluorolidocaine as competition ligands (Fig. 1b) revealed that the novel lidocaine derivative provided the identical level of non-specific binding (B_NS_) as the established drug.

As a consequence, both saturating concentrations of lidocaine and fluorolidocaine can be used to determine B_S_, which provides a major advantage for *in vivo* experiments, allowing to use lidocaine as a tried and tested clinical drug. In addition, we performed dose-dependent displacement assays (Figure S3) and found that both molecules exhibit very similar low double digit micromolar IC_50_ values. Thus, both molecules are not only mutually exclusive for receptor sites in the myocardium but also engage with SCN5A with equal affinity.

To ensure that our protocol represents binding equilibrium conditions, thus allowing to make quantitative comparisons, we varied incubation times (Fig. 1c) and determined that BS reached a constant level after only a 5 min incubation. Using the DeBlasi considerations for determination of receptor numbers, B_S_ directly correlates to the receptor density B_max_ and can thus be used for quantitative comparison of channel populations^18^. Therefore, radiocaine measurements could be used to compare SCN5A expression levels.

Similar to rapid kinetics towards binding equilibrium, the time-course of wash-out was fast (Fig. 1d) with a 50% reduction of total binding after 1 min. The fast time course of association and dissociation observed in radiocaine autoradiography matches the instant antiarrhythmic effect of a lidocaine infusion, which quickly ceases after the infusion is stopped^15^.

### Radiocaine mPET imaging in rats

**Figure 2:**
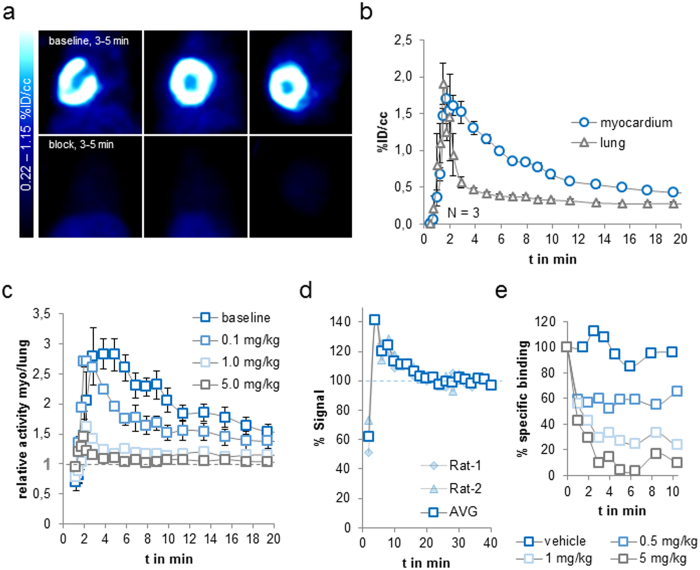
*In vivo* radiocaine mPET-imaging using healthy rats. (**a**) Summed images (3–5 min) of rat myocardium after a i.v. bolus injection of 750 μCi radiocaine (top) or a 5 mg/kg dose i.v. lidocaine dissolved in saline five minutes before 1036 μCi radiocaine (bottom) (baseline animal was injected with an equivalent volume of saline). Images depict coronal, sagittal and transverse views of the rat thorax. (**b**) Time-activity-curves (TACs) of rat myocardium and lung (N = 3, error bars represent ± one Stdev). (**c**) Ratio of myocardium to lung over time and as a function of drug occupancy. Doses of 0.1, 1.0 and 5.0 mg/kg lidocaine or an equivalent volume of saline were injected i.v. 5 min before radiocaine injection (N = 3 for baseline, N = 2 for each dose, error bars represent ± one Stdev, two-way ANOVA analysis with replication and an alpha value of 0.001 delivered p = 4.04^−22^, allowing to conclude that the myocardium/lung ratio changes with ≥99.9% certainty as a function of drug occupancy). (**d**) Bolus + infusion paradigm. Radiocaine was injected i.v. followed by continuous syringe-pump infusion for 40 min (rate = 1 ml/hour). The signal represents the ratio of myocardium over lung and is normalized to the plateau after 20 min. (**e**) Bolus + Infusion paradigm with in-scan lidocaine challenge. Using an additional i.v. line, vehicle or increasing concentrations of lidocaine (0.5–5 mg/kg) were injected. Specific binding is calculated as the myocardium/lung ratio at the TAC-plateau minus the same ratio after injection of a 5.0 mg/kg lidocaine dose. Each trace represents one animal (N = 4).

In order to investigate to what extent the *in vitro* specific binding correlates to *in vivo* specific binding and thus test the potential of radiocaine as an *in vivo* PET radiotracer for SCN5A, we performed cardiac mPET experiments in healthy rats (Fig. 2).

We employed a classical single bolus injection paradigm, which provided a full representation of the rat myocardium from coronal, sagittal and transverse views (Fig. 2a). Despite the movement from heart contraction and breathing, the non-motion-corrected images gave a clear view of the myocardium surrounding the left ventricle; horseshoe-shaped in the coronal view or circular in sagittal- and transverse views. The myocardial baseline signal was strong with up to 2% ID/cc and the time-course of radiotracer binding was fast, in line with our *ex vivo* experiments (Fig. 2b). Advantageously, clearance from the blood pool was even more rapid, which was a prerequisite for *in vivo* imaging in order to minimize partial volume effects from the surrounding lung tissue, which has no expected specific binding, but represents radioactivity in the blood pool. Likewise, cardiac PET imaging is often hampered by partial volume effects due to radiotracer and/or radioactive metabolites accumulating in the liver. Due to the fast time course of radiocaine binding in the myocardium, these effects did not affect the signal since liver uptake occurred on a slower time course.

Motivated by advantageous radiocaine pharmacometrics with very rapid blood clearance, fast myocardial binding and slower uptake in the liver, we further investigated the nature of the myocardial signal *in vivo*. In order to get an appreciation for the volume of distribution between the blood and myocardium, we used the ratio of the myocardial signal to the lung signal over time. Given the small volume of the heart compared to the mPET resolution and the expected myocardial uptake, it was advantageous in rats to use the lungs as a reference for blood activity instead of the left ventricle. Using the ratio of the myocardium as the expected binding region to the lung as a reference region for the blood signal provided several advantages. Firstly, potential differences in free radiotracer between animals could be neglected, secondly, the total injected dose was not needed for analysis, and thirdly, potential differences in attenuation were crossed out. As a consequence, the variance between animals and experiments was very small, demonstrating the robustness of the signal and allowing us to use a comparatively small sample size.

We found that the myocardium to lung ratio peaked at approximately 3 after 3–5 min and clears back to “one” over the course of 20 min (Fig. 2c). To further interrogate the specificity of the signal, and the sensitivity of the myocardium to lung ratio towards available SCN5A, we administered increasing concentrations of lidocaine intravenously 5 minutes prior to radiocaine injection (Fig. 2c). Compared to vehicle injection, signal reduction was observed starting at doses of 0.1 mg/kg lidocaine, corresponding to a 5.8 μM concentration assuming a blood volume of 30 mL (according to blood volume BV = 0.06 × body weight BW + 0.77)^19^. The lower panel of Fig. 2a shows complete block of the myocardial radiocaine signal, which is represented by a signal to background ratio of 1 for a 5.0 mg/kg lidocaine dose in Fig. 2c. Interestingly, the ratio of myocardium to lung activity highlights the effect of drug occupancy already in the first minutes after tracer injection, demonstrating that even very early signal represents specific binding. Using *in vivo* lidocaine competition experiments we were thus able to show that the myocardial radiocaine signal is fully saturable and the extent of specific binding is even higher for the *in vivo* mPET experiment than for the *ex vivo* autoradiography. Furthermore, our experiments demonstrate that the ratio of myocardium to lung signal allows comparing available SCN5A.

We next performed bolus-infusion experiments to investigate the radiocaine signal at equilibrium conditions, eliminate the factor “flow” and apply in-scan challenges (Fig. 2d). Applying an intravenous bolus of ~150 μCi radiocaine followed by a constant infusion of ~300 μCi through the same vein over the course of 60 min provided a stable baseline signal of the ratio of myocardium/lung already after 20 min. Injection of vehicle through an additional i.v. line led to no change in this signal (Fig. 2e) despite the increase in blood volume. However, lidocaine injections reduced the radiocaine signal in a dose-dependent manner confirming reversible radiocaine binding and competition for lidocaine binding sites. A complete reduction to background levels and a myocardium/lung ratio of one was achieved with 5 mg/kg lidocaine, in line with our pre-block experiments (Fig. 2c). These in-scan challenges allowed us to estimate the *in vivo* IC_50_ of lidocaine at the myocardium of ~0.7 mg/kg. Applying the same assumptions for blood volume as stated above, this corresponds to a 40 μM concentration, which is in the expected range for lidocaine^9^.

In addition, the time course of the reduction in radiocaine signal allowed us to estimate the residence time (t_1/2_^−1^) of the radiotracer to ~1 min^−1^, which is in line with the fast wash-out in autoradiography and the quickly ceasing efficacy of an i.v. lidocaine infusion in patients. Interestingly, the plateau of the full block with a 5 mg/kg lidocaine dose was reached 3 minutes after the pharmacological dose, which is the same time that the radiocaine bolus needs to reach its peak signal (Fig. 2c), indicating that radiocaine and lidocaine have very similar binding kinetics.

### Radiocaine PET imaging in non-human primates

**Figure 3:**
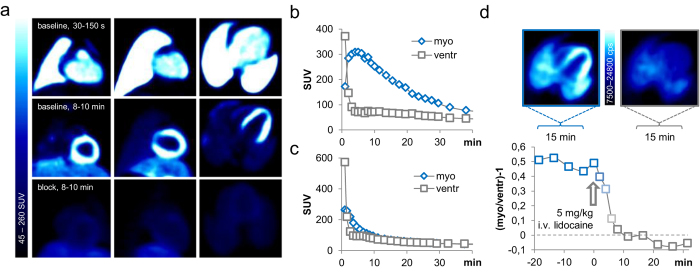
Radiocaine PET-imaging in non-human primates. (**a**) Thoracic PET-images of a baboon injected with 5.08 mCi radiocaine. Upper panel shows summed images (30–150 seconds) from coronal, sagittal and transverse view with blood-filled heart and lungs. The middle panel shows the same scan summed from 8–10 min with a clear myocardial signal. The lower panel shows the same animal in a radiocaine scan treated with 5 mg/kg i.v. lidocaine 5 minutes prior to tracer injection. (**b**) TACs of the myocardium and ventricle of the baboon shown in the upper panel of (**a**). The larger organ (compared to rat) allowed using the ventricle as an internal reference for the radiocaine blood signal. (**c**) Analogous TACs of the baboon shown in the lower panel of (**a**). The competition ligand lidocaine, injected 5 minutes prior to radiocaine suppresses the myocardial signal to background levels. (**d**) An additional baboon investigated with a bolus + infusion paradigm and an in-scan lidocaine challenge. A dose of 5.0 mg/kg lidocaine was injected after the myocardial signal had reached a plateau. 10 min after this drug challenge, the myocardial signal was reduced to background levels.

To determine the potential of radiocaine for translational imaging, we investigated the extent of signal specificity and background clearance in PET-imaging experiments with two non-human primates (Fig. 3). Single bolus injection of radiocaine in a healthy baboon provided a full representation of the myocardium (Figs 3a and S4). At early time points (30–150 sec), the thoracic PET scan showed the blood-filled heart and lungs. Already after 2–3 min, the blood background cleared and the left myocardium appeared as a strong signal with up to 300 SUV. Administering a 5 mg/kg dose of lidocaine 5 min prior to radiocaine injection blocked the myocardial signal (Fig. 3a), allowing us to conclude that the radiocaine signal at the myocardium of non-human primates was also specific. Figure 3b and c show the time activity curves of the left myocardium (blue) and ventricle (white) obtained from the baseline and lidocaine competition experiments described above. The larger organ (compared to rat) allowed us to utilize the left ventricle as a reference for blood radioactivity. Interestingly, the signal of myocardium peaks at ~3-fold over the blood signal and can be fully blocked to blood levels by 5 mg/kg lidocaine, in analogy to our rodent experiments.

We confirmed specific binding in a second healthy baboon using a bolus + infusion paradigm. Using a K_Bol_ of 90 min in a 90 min scan, we injected an i.v. dose of 5 mg/kg lidocaine (through a second i.v. line) after equilibrium conditions were reached. Within 10 minutes, the myocardial signal had reached the background level of the ventricle, allowing to estimate a residence time of 5 min^−1^. The overall time-course of binding in both bolus and bolus + infusion experiments was longer in baboons than in rats, which was expected for the higher order species. However, scan-times of only 20–30 min were sufficient to capture the majority binding kinetics and will be a major advantage for human imaging, in particular with the ventricle or the lungs as an internal quantitative reference.

Taking advantage of the larger organ (compared to rats) and guided by magnetic resonance imaging (MRI) we deduced further anatomical information of radiocaine binding in the heart using image fusion from different times after injection (Figure S4). A summed PET image from 30–90 seconds after radiotracer injection expectedly showed no myocardial signal but provided a reference for the blood volume in different compartments in the heart. Both atria and the left ventricle were clearly visible from coronal view as well as the right and left ventricles from transverse view. Summed PET images from later time points (90–210 sec, Figure S4) demonstrated uptake in the myocardium of the atria and the right ventricle (RV) in addition to the dominant LV, however with much lower intensity. Image fusion of the blood and myocardial signals confirmed this assignment. The dominant signal of radiocaine at the LV compared to the atria provides a molecular explanation for the efficacy of lidocaine and related compounds for treating ventricular, but not atrial fibrillations. The relative signal intensity, and thus SCN5A density, between LV, RV and atria might be an interesting assessment of heart health and a function of disease in clinical imaging.

### Radiocaine autoradiography in human heart explants

**Figure 4:**
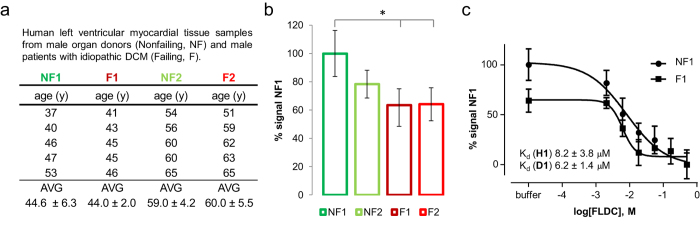
Radiocaine autoradiography with explanted human heart tissue. (**a**) Summary of investigated subjects; NF = non-failing, F = failing (idiopathic dilated cardiomyopathy, DCM). (**b**) Comparison of specific radiocaine signal relative to group NF1 determined by autoradiography at equilibrium conditions with 500 μM lidocaine as blocking agent (N = 5, n ≥ 2, significance * is defined by p < 0.05 as determined by student t-test, error bars represent ± 1 stdev). (**c**) Dose-response experiments with increasing concentrations of fluorolidocaine. Using self-displacement, the IC_50_-value generated with this assay equals the K_d_-value. (N = 5, n = 1, error bars represent ± 1 stdev).

With a well-behaved and validated translatable tool, we investigated SCN5A expression differences in healthy and diseased human left ventricular tissue samples (Fig. 4). To accomplish this, we used autoradiography and compared non-failing human donor heart samples to explanted tissue from patients who had suffered from heart failure due to idiopathic dilated cardiomyopathy (DCM) and required cardiac transplantation (Fig. 4)^20^.

Our tissue samples comprised a moderately sized group of age and gender matched subjects (Fig. 4a). NF1 and F1 represent five subjects each with an average age of ~44 years. Groups NF2 and F2 also included five subjects each around 60 years of age. In order to detect changes is SCN5A density, we applied autoradiography conditions previously established for rat myocardium (15 min incubation, 1 min wash, Fig. 1) and used lidocaine as the competition ligand at 500 μM concentration. We found a ~30% decrease of B_max_ between NF1 and F1 (Fig. 4b). To the best of our knowledge, this is the first time that cardiac sodium channel density is linked to heart failure in humans. Even though not significant in our sample groups, there seems to be a trend between the two healthy groups NF1 and NF2 as signal reduces with age. Since the prevalence of arrhythmias, conduction disturbances, and heart failure all increase with age, it will be interesting to measure how the radiocaine-SCN5A signal changes in longitudinal studies of the aging process^21^.

To exclude that the reduced signal was the result of a decrease in affinity in diseased vs. healthy tissue and confirm a deficit in channel density, we performed dose-response experiments with fluorolidocaine as the competition ligand. Using the modified Cheng-Prusoff equation for self-inhibition, these measurements allowed us to determine K_d_-values for the groups NF1 and F1 (Fig. 4c)^18^. We found that in average there was no difference in Kd-values between the groups and again observed a ~30% reduction in Bmax. We conclude from these data that the sodium channel density was reduced in our tissue samples of DCM-failing myocardium compared to non-failing, age-matched tissue. Given the well matched binding properties between our *in vivo* and *ex vivo* experiments, we therefore think that radiocaine will not only be a powerful preclinical research tool, but will also have an impact on clinical human imaging, allowing measurements of SCN5A density in the heart and relate this signal to disease.

In summary, we developed a novel radiotracer – radiocaine – that enables for the first time *in vivo* imaging of the cardiac sodium channel SCN5A. Our novel tool allows measuring *in vivo* channel density and drug occupancy levels in rats and baboons. In addition, we have demonstrated reduced SCN5A signal by autoradiography in myocardial tissue from failing human heart explants, which might be a future diagnostic marker. To our knowledge, there is currently no therapy or treatment that may increase channel density and radiocaine imaging might facilitate development of such drugs or identify beneficial lifestyle changes. Since radiocaine is based on the well-established anti-arrhythmic drug lidocaine, we envision progression to human imaging in the near future, allowing us to link changes in SCN5A signal to diseases of the heart. Using the clinically established isotope F-18 will allow centralized production and distribution, facilitating a wide-spread use of our tool. Given the fundamental involvement of SCN5A in heart function, we envision a broad range of applications in cardiac imaging in addition to cardiomyopathies. For example, mutations in the *SCN5A* gene have been implicated in both long QT syndrome and Brugada syndrome, yet the determination of which patients are at risk for sudden cardiac death from arrhythmias and need a prophylactic implantable cardioverter-defibrillator is an area of clinical uncertainty^22^. The ability to measure SCN5A density in living human hearts using PET imaging and the radiotracer radiocaine may allow for individualized molecular imaging that uncovers the underlying pathophysiology of their cardiovascular diseases. Eventually, this imaging technique may serve as a potent diagnostic tool to stratify arrhythmia risk in patients with cardiomyopathies and determine the need for treatments such as antiarrhythmic medications, pacemakers, and implantable cardioverter-defibrillators. Our lab is dedicated to expanding the reach of radiocaine to further applications in cardiovascular, neuroscience and pain research.

## Additional Information

**How to cite this article**: Hooker, J. M. *et al*. Imaging cardiac SCN5A using the novel F-18 radiotracer radiocaine. *Sci. Rep.* **7**, 42136; doi: 10.1038/srep42136 (2017).

**Publisher's note:** Springer Nature remains neutral with regard to jurisdictional claims in published maps and institutional affiliations.

## Supplementary Material

Supplementary Information
