# Supplementary material for: Imaging cardiac SCN5A using the novel F-18 radiotracer radiocaine
Source: Sci Rep. 2017 Feb 16;7:42136. doi: 10.1038/srep42136 (PMC5311962; doi:10.1038/srep42136)
Supplement: Supplementary Information [file srep42136-s1.pdf]

Imaging cardiac SCN5A using the novel F-18 radiotracer radiocaine

Jacob Hooker[1], Martin A Strebl[1], Frederick A Schroeder[1], Hsiao-Ying Wey[1], Amrut V Ambardekar[2], Timothy A. McKinsey[2], Matthias Schoenberger[1,3*]

[1] Harvard Medical School, Massachusetts General Hospital, Martinos Center for Biomedical Imaging, 149 13th Street, 02129 Charlestown, MA, USA, [2] Department of Medicine, Division of Cardiology and Consortium for Fibrosis Research and Translation, University of Colorado – Anschutz Medical Campus, Aurora, CO, USA, [3] current address: KU Leuven, Department of Pharmaceutical and Pharmacological Sciences, Herestraat 49, Leuven, Belgium

SUPPLEMENTARY INFORMATION

Supporting Figures

Figure S1. Chemical design page 2

Figure S2. Synthesis scheme page 3

Figure S3. IC_{50} determination page 4

Figure S4. PET image fusion page 4

Supporting Methods

Radiochemical Synthesis page 5

Analytical HPLC page 5

Semipreparative HPLC page 6

Radiotracer formulation page 6

Chemical Synthesis page 6

Abbreviations

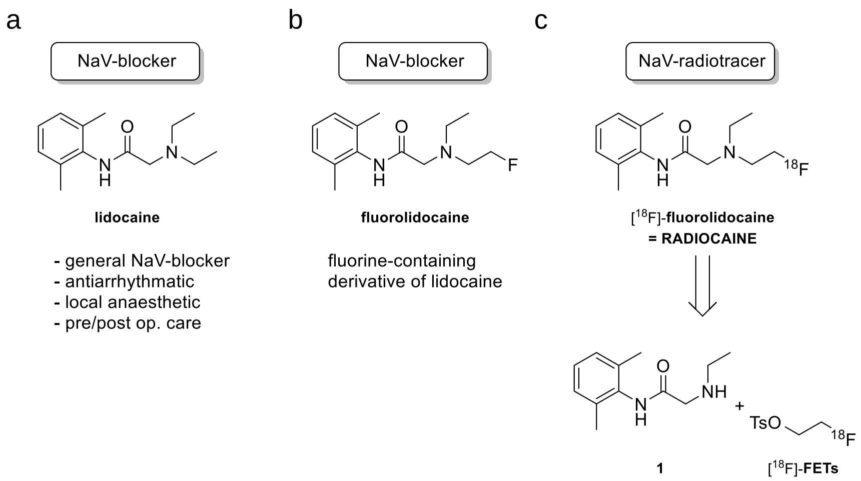

Figure S1. Chemical design a) Chemical structure of lidocaine, which is a clinically used general NaV-blocker. Its main applications are treatment and prevention of ventricular fibrillations as well as analgesia. b) Structure of a fluorine-containing lidocaine derivative "fluorolidocaine". The *N,N*-diethyl functional group of lidocaine is very tolerant toward structural modifications. Many known analgesics are designed around variations of this motif. Fluorolidocaine, inspite being a novel structure, is designed to have minor or no pharmacological differences to lidocaine. Therefore, we formally exchanged a proton for a fluorine atom at a structurally very tolerant position. c) [^{18}F]-fluorolidocaine is an isotope of fluorolidocaine with the positron emitting radioisotope F-18. F-18 is a widely used isotope for clinical PET-imaging with a half-life of 109 min. We planned to synthesize using secondary amine 1 and the know F-18 prosthetic group [^{18}F]-fluoroethyltosylate ([^{18}F]-FETs) in a conceptually simple nucleophilic substitution. We call the PET-radiotracer [^{18}F]-fluorolidocaine "**radiocaine**".

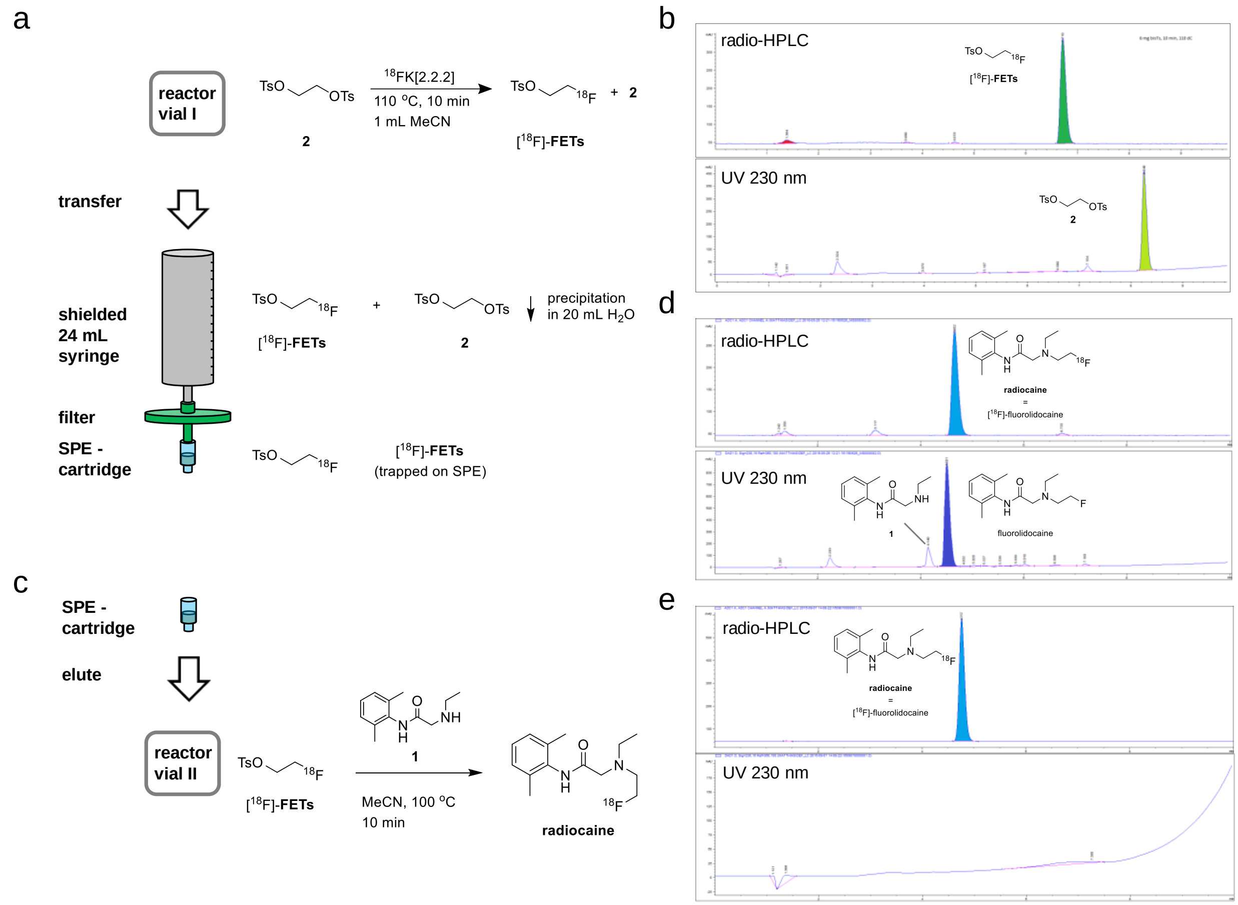

Figure S2. Scheme of radiocaine synthesis and chromatographic analysis. a) Synthesis and isolation by precipitation/filtration of [^{18}F]-fluoroethyltosylate ([^{18}F]FETs). b) Analytical HPLC chromatogram of the reaction mixture in reactor vial I after 10 min. c) Alkylation of **1** with ([^{18}F]FETs) yielding radiocaine. d) Analytical HPLC chromatogram of the reaction mixture in reactor vial II with addition of a fluorolidocaine standard. e) Analytical HPLC chromatogram of isolated and formulated radiocaine.

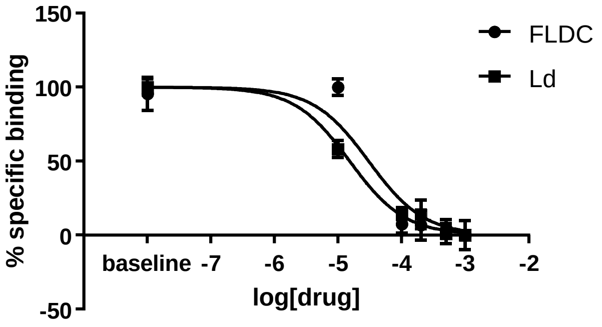

Figure S3. IC_{50} determination of fluorolidocaine (FLDC) and lidocaine (Ld). Rat heart myocardial slices were used for radiocaine autoradiography using the described protocol. 5 slices from 2 donor animals were incubated with vehicle or increasing concentrations of either lidocaine or fluorolidocaine. Specific binding was determined as the respective signal minus the signal at the highest concentration of competition ligand and values were normalized to the vehicle signal (baseline). Data were analyzed and fitted using Graphpad Prism ® software, error bars indicate ± one stdev. The 95% confidence interval for the IC_{50} of FLDC is 20 – 43 μM and 12 – 20 for μM for Ld.

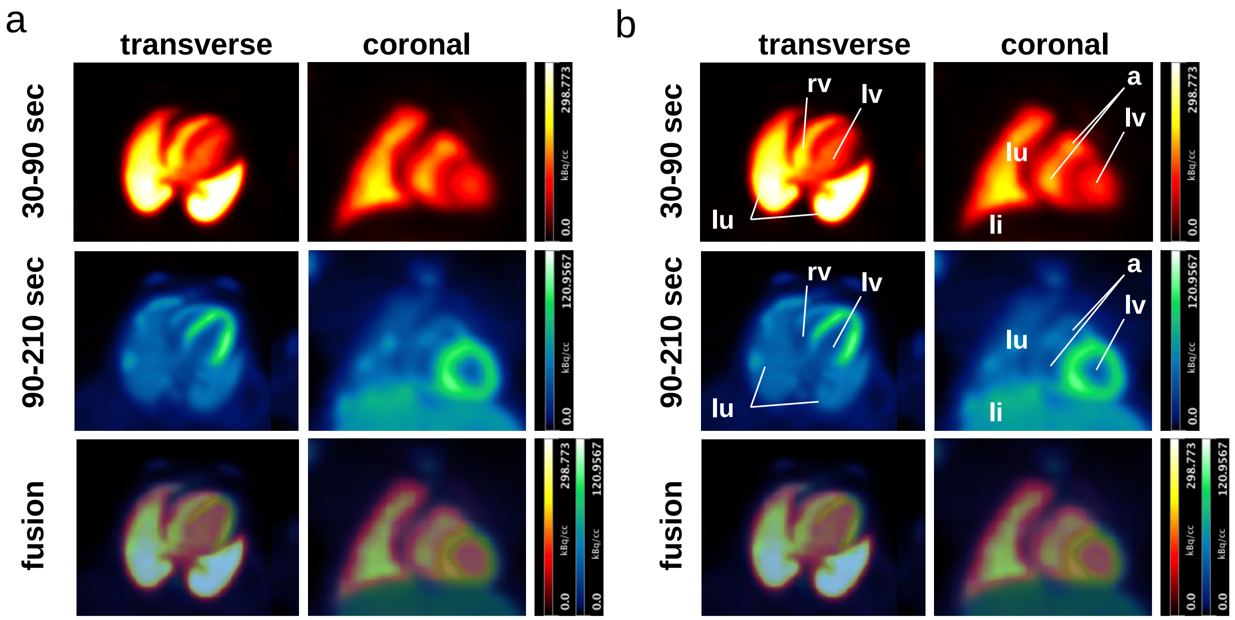

Figure S4. PET image fusion of non-human primate injected with radiocaine. a) Transverse view from early times (30-90 sec) shows lungs, and both ventricles filled with blood. Coronal view shows lung, both atria and the left ventricle filled with blood. At later times (90-120sec), the signal from the myocardium surrounding the left ventricle appears as the strongest signal and the myocardium from the right ventricle (transverse view) as well as the atria (coronal view) is also visible. The fusion image of the blood signal and myocardial signal highlights the different compartments and the surrounding muscle. b) Same images as a) with annotations: lu = lung, rv = right ventricle, lv = left ventricle, li = liver, a = atria. Images were analyzed using PMOD ® 3.8 software.

Radiochemical synthesis

The radiocaine synthesis proceeded in a two-step process, which included synthesis of [^{18}F]-fluoroethyltosylate ([^{18}F]FETs) and subsequent alkylation of secondary amine **1**. First, 5 mg of bis-tosylate **2** were reacted with freshly dried ^{18}FK[2.2.2] in 1 mL MeCN for 10 min at 110 ^{o}C (Figure S1a). Monitoring this reaction using analytical HPLC with UV and radiation detection showed that nearly all F-18 (red peak, radio-HPLC) had reacted with **2** (light green, UV 230 nm) to [^{18}F]FETs (dark green, radio-HPLC) (Figure S2b). To separate the F-18 prosthetic group [^{18}F]FETs from precursor **2**, the poor solubility of **2** in water was exploited as well as the sufficient solubility of [^{18}F]FETs at trace concentrations combined with its chemical stability towards hydrolysis. Adding the reaction mixture from reactor vial I to a shielded 24 mL syringe with 20 mL of water led to precipitation of **2**. This precipitate was held back by a 22 um filter and the dissolved [^{18}F]FETs was trapped on a strataX reversed phase cartridge. After disconnection from the filter, the column was dried with air using a fresh 24 mL syringe and eluted in 500 μL MeCN. This process typically yielded [^{18}F]FETs in 40-60% non-decay corrected isolated yield (for example, 98 mCi [^{18}F]FETs from 205 mCi F-18). Freshly prepared [^{18}F]FETs dissolved in 500 μL MeCN was added to a new reactor vial containing 2 mg of precursor **1** (Figure S2c). Heating this mixture to 100 ^{o}C for 10 min yielded radiocaine. Figure S2d shows the analytical HPLC chromatogram of this reaction mixture. Nearly all [^{18}F]FETs had reacted to a new radioactive peak (light blue, radio-HPLC). Addition of a fluorolidocaine standard (dark blue, UV 230 nm) to this mixture revealed that this new radioactive peak was radiocaine. The reaction mixture from reactor vial II was purified using semi-preparative HPLC yielding pure and injectable radiocaine. Panel e) shows an analytical HPLC chromatogram of isolated and formulated radiocaine with ≥ 99.5% radiochemical purity. The isolated, non-decay corrected radiochemical yield of this reaction typically ranges from 40-60% (for example, 41 mCi radiocaine from 93 mCi [^{18}F]FETs). The specific activity of radiocaine was determined using an analytical HPLC calibration curve. At the time of injection, specific activity was 4.9 ± 3.5 mCi/nmol in our experiments.

Analytical HPLC conditions

Solid phase: Agilent Eclipse XDB-C18 (5 μm 4.6 x 150 mm)

Mobile phase: A = 0.1% TFA in water, B = 0.1% TFA in MeCN. Gradient: 0-1 min (5% B), 1-5 min (5-50% B), 5-6 min (50% B), 6-10 min (50-95% B)

Flow: 1.5 ml/min

R_t (bis-tosylate 2) = 8.25 min

R_t ([^{18}F]FETs) = 6.71 min

R_t (precursor 1) = 4.14 min

R_t (fluorolidocaine) = 4.60 min

R_t (radiocaine) = 4.65 min

Semipreparative HPLC conditions

Solid phase: Phenomenex Luna 5u C8(2) (100A 10 x 250 mm)

Mobile phase: 8% EtOH (200 proof) in water + 0.01% phosphoric acid

Flow: 4.0 ml/min

R_t (precursor 1) = 7 – 9 min

R_t (radiocaine) = 17 – 18 min

Radiotracer formulation

The radiocaine peak isolated from semipreparative HPLC (typically 4 – 6 mL) was diluted with a 1/10 volume of 10 x PBS buffer and filtered through a sterile 22 μm filter into a 10 mL sterile injection vial. This injectable stock solution was diluted with sterile saline to adjust for the desired volume and amount of radioactivity. Typically, concentrations of 1 mCi/mL and volumes of 1 mL/kg were injected in rats (e.g. 0.5 mCi in 0.5 mL for a 500 g animal). For non-human primate imaging, doses of ~5 mCi were injected in volumes of 4 – 6 mL.

Chemical Synthesis of fluorolidocaine

Secondary amine **1** (500 mg, 2.4 mmol, 1.0 eq) was dissolved in 5 mL DMF. Fluoroethyltosylate (635 mg, 2.9 mmol, 1.2 eq) were added, followed by potassium carbonate (440 mg, 5 mmol, 2.1 eq). The resulting suspension was stirred at 90 ^oC for 3 hours. After cooling to room temperature, the reaction mixture was diluted with 100 mL ethyl acetate abd washed with 25 mL 1-M-$NaOH_{(aq)}$ (1x) and 25 mL of 1:1 mixture of brine and 1-M-$NaOH_{(aq)}$ (3x). The organic layer was dried using sodium sulfate and concentrated using rotary evaporation yielding an oily residue. This crude mixture was purified using automated flash chromatography (ISCO, 24 g silica column, gradient 0 – 5% MeOH in dichloromethane) yielding rediscovered starting material **1** (240 mg, 48%) and the disered product (335 mg, 1.3 mmol, 56%). Fluorolidocaine was isolated as an oil, which solidified to an off-white solid upon storage at room temperature.

TLC (silica, 5% MeOH/DCM, UV): single spot, Rf = 0.41

LRMS (LCMS, ESI): single peak, $(M+H^+)_{(calc)}$ = 253.2; $(M+H^+)_{(found)}$ = 253.2

HRMS (ESI): $(M+H^+)_{(calc)}$ = 253.1711; $(M+H^+)_{(found)}$ = 253.1743

^{13}C-NMR (125 MHz, acetonitrile-d_3, chemical shift in ppm): 135.65; 134.72; 127.84; 126.89; 82.03 (d, ^1J = 164 Hz); 57.25; 54.32 (d, ^2J = 20 Hz); 49.64; 17.73; 11.24

Abbreviations

DMF	dimethylformaide
FETs	fluoroethyl tosylate
HPLC	high pressure liquid chromatography
HRMS	high resolution mass spectrometry
LRMS	low resolution mass spectrometry
MeCN	acetonitrile
PET	positron emission tomography
PBS	phosphor buffered saline
R_t	retention time
Stdev	standard deviation
TLC	thin layer chromatography
